# Articular and Peripheral Nervous System Involvement Are Linked to the Long-Term Outcome in Primary Sjögren's Syndrome: The Relevance of Single Organ Manifestations Rather Than a Composite Score as Predictors

**DOI:** 10.3389/fimmu.2019.01527

**Published:** 2019-07-10

**Authors:** Luca Quartuccio, Saviana Gandolfo, Alen Zabotti, Sara Zandonella Callegher, Cinzia Fabro, Salvatore De Vita

**Affiliations:** Department of Medical Area (DAME), Rheumatology Clinic, University of Udine, Udine, Italy

**Keywords:** primary Sjögren's syndrome, extraglandular involvement, disease activity, peripheral nervous system, ESSDAI

## Abstract

**Objective:** The disease course in primary Sjögren's Syndrome (pSS) differs in different subsets of patients. The aim of this study was to clarify whether the pattern of organ involvement may improve the prediction of the very long-term disease outcome.

**Methods:** We collected the data of 255 patients. The total European League Against Rheumatism (EULAR), EULAR Sjögren's Syndrome Disease Activity Index (ESSDAI) score was compared with the pattern of organ involvement, as differentiated by the single ESSDAI domains: (i) at disease diagnosis, and (ii) in the follow-up, by verifying the appearance of new ESSDAI domains and/or the worsening of already active ESSDAI domains.

**Results:** The mean follow-up duration was 9.1 ± 6.9 years. At disease diagnosis, only the articular activity at baseline could predict the long-term outcome of pSS detected at last follow-up visit, being protective in terms of stable or improved disease activity, as measured by ESSDAI [OR 2.9 (1.6–5.4), *p* = 0.01]. In the follow-up, the onset, and/or worsening of either the peripheral nervous system (PNS) domain (by multivariate and univariate analysis), or the biological domain (only by univariate analysis) correlated with a higher disease activity at the last visit [PNS domain: OR 5.9 (2.4–14.5), *p* < 0.0001; biological domain: OR 1.9 (1.0–3.8), *p* = 0.043]. A significantly higher number of patients with articular involvement were taking hydroxychloroquine at the last follow-up visits, if compared with patients without (41/130, 31.5 vs. 13/125, 10.4%, *p* < 0.0001).

**Conclusion:** Single organ disease manifestations of SS, herein identified as the articular, PNS and biologic involvement, are relevant to predict the very long-term outcome in pSS.

## Introduction

The spectrum of primary Sjögren's syndrome (pSS) manifestations can vary from a sicca syndrome, fatigue, extraglandular disease, and non-Hodgkin's lymphoma (NHL) development, (1–5). In 15% of patients, especially those with an immunological profile suggestive of B-cell activation, severe systemic manifestations are present, as recently described by Baldini et al. (6).

The European League Against Rheumatism (EULAR) task force on pSS has developed and validated a disease activity index for pSS: the EULAR Sjögren's Syndrome Disease Activity Index (ESSDAI) used both in clinical trials and in daily practice (7–9). However, as recently highlighted for lymphoma evolution, the pattern of organ involvement or specific biomarkers may be more relevant for outcome prediction in pSS than a composite score as ESSDAI (10). Furthermore, novel drugs can be better applied when considering the pattern of organ involvement in pSS, rather than the whole score of disease activity as measured by ESSDAI (11). Thus, the aim of this study was to answer two important clinical questions at disease diagnosis or during its follow-up; first, if the pattern of organ involvement at baseline (as for instance dissected by the single ESSDAI domains) can predict the level of disease activity at the last follow-up; secondly, if there are particular disease manifestations, appearing *ex-novo*, or worsening during the follow-up, which can predict the same long-term outcome. To this end, a monocentric cohort of 255 pSS patients with a long follow-up was analyzed.

## Methods

### Patients

We conducted an observational retrospective study collecting the data of 255 consecutive patients suffering from pSS who referred to our Center (Rheumatology Clinic, University Hospital of Udine, Italy) from January 2001 to April 2018. All patients fulfilled American-European Consensus Group (AECG) 2002 criteria; exclusion criteria were: hepatitis C virus/HIV infections, past head and neck radiation treatment, sarcoidosis, graft vs. host disease, current use of anticholinergic drugs, or associated systemic autoimmune diseases (12).

Data collected included: ethnicity, gender, age at diagnosis, and at last follow-up, presence or absence of dry eye and dry mouth symptoms, hematological neoplasia (number and histological type), diagnostic tests for pSS (ocular tests, oral tests, and salivary gland biopsy according to the recommendations of the European Community Study Group) (13, 14), and laboratory tests (ANA determined by indirect immunofluorescence; anti-Ro/SSA and anti-La/SSB antibodies determined by commercial kits for the ELISA tests; rheumatoid factor (RF) detected by nephelometry; C3 and C4 levels determined by immunoturbidimetry and cryoglobulins detected by cryoprecipitation).

### Definition of Systemic Involvement

Systemic involvement was defined according to the ESSDAI, which evaluates 12 organ by organ domains. Clear clinical definition of the single domains was reported elsewhere (7). Each domain is divided into three or four levels according to the degree of activity and scored as 0 (no activity), 1 (low activity), 2 (moderate activity), or 3 (high activity). These scores are then multiplied by an assigned weight factor, ranging from 1 to 6, with the total score ranging from 0 to 123 points (7). Low disease activity is considered if ESSDAI score is <5; moderate activity if ESSDAI score = 5–13, and high activity if ESSDAI score is ≥14 (15). In this study, we considered all 12 domains, the 10 clinical and the two laboratory domains. We calculated baseline ESSDAI scores (i.e., calculated at the diagnosis), ESSDAI scores at last follow-up visit and the cumulative ESSDAI score (i.e., the sum of the maximum scores per each domain achieved at any time during the follow-up period), as previously performed in other studies (16, 17), by verifying the new onset ESSDAI domain and/or the worsening in already active ESSDAI domains in the whole available follow-up.

### Statistical Analysis

Categorical variables are presented as frequencies and percentages. Quantitative variables are described with the mean ± standard deviation (SD) or with the median (range). The odds ratio (OR) was expressed with 95% CI. Chi square test was used to assess differences between subgroup of patients, and mean differences were expressed with 95% CI. In the case of distributions with extreme outliers a non-parametric Wilcoxon signed-rank test for paired data was used. In order to identify possible predictive factors of long-term outcome, univariate (by Chi square tests) and multivariate analyses (by stepwise regression analysis) were conducted. All significance tests were two-tailed and values of *p* < 0.05 were considered significant. Prediction model was developed by considering the following independent variables: demographic data, baseline data regarding the presence of RF, cryoglobulins, anti-Ro/SSA, anti-La/SSB antibody, low levels of C3 and C4, along with each ESSDAI domain (considered as dicotomic variable “presence/absence”). All patients were included in the analysis.

## Results

### Baseline Characterization

Our cohort included 255 pSS patients, 236 women (92.5%), and 19 men (7.5%). The mean age at diagnosis was 52 (13) years (range 10–84 years). The observation period covered a mean time of 9.1 (6.9) years from the diagnosis to the last follow-up visit.

At diagnosis 245/255 (96%) patients presented with ocular symptoms, 248/255 (97.2%) with oral symptoms, 214/255 (84%) had abnormal ocular tests, and 105/135 (77%) abnormal oral diagnostic tests; 106/127 (83%) patients presented a positive minor salivary gland biopsy. The frequency of immunological markers at diagnosis was as follows: positive ANA (>1:160) in 217/255 (85%) patients, anti-Ro/SSA antibodies in 195/255 (76.5%), anti-La/SSB antibodies in 126/255 (49.4%), RF was positive in 115/255 (45%), low C3 levels in 56/255 (22%), low C4 levels in 40/255 (16%), and positive serum cryoglobulins in 22/255 (8.6%). The baseline characterization of patients is summarized in Table 1.

**Table 1:** Baseline characteristics.

**Features**	***N* = 255**
Age at diagnosis, years, *mean ± SD*	52 ± 13
Age at last follow-up, years, *mean ± SD*	61 ± 13
Gender, female, *n (%)*	236 (92.5%)
Ethnicity, white, *n (%)*	255 (100%)
Ocular symptoms, *n (%)*	245 (96%)
Oral symptoms, *n (%)*	248 (97%)
Positive salivary gland biopsy, *n (%)*	106/127 (83%)
Focus score, *mean ± SD*	1.79 ± 1
Anti-Ro/SSA positive, *n (%)*	195 (76.5%)
Anti-La/SSB positive, *n (%)*	126 (49.4%)
ANA positive >1:160, *n (%)*	217 (85%)
RF positive, *n (%)*	115 (45%)
Cryoglobulin positive, *n (%)*	22 (8.6%)
Low C3 levels (<90 mg/dl), *n (%)*	56 (22%)
Low C4 levels (<10 mg/dl), *n (%)*	40 (16%)
B-cell lymphoma at baseline, *n (%)*	18 (7%)
B-cell lymphoma during follow-up, *n (%)*	12 (4.7%)

*RF, rheumatoid factor; ANA, antinuclear antibodies*.

### Baseline Disease Activity

At baseline the median ESSDAI score was 4 (range 0–31). The disease activity was low in 140/255 (55%) patients, moderate in 84/255 (33%), and high in 31/255 (12%). The total amount of active ESSDAI domains at baseline was 508. The most frequent active domains were: articular (130/508, 26%), biological (125/508, 25%), glandular (77/508, 15%), hematological (58/508, 11%). Thirty-six out of two hundred and not hundreds and fifty-five (14%) patients had no systemic involvement (i.e., baseline ESSDAI = 0), and 21/36 (58%) of these patients still lacked any activity at the end of the follow-up. Baseline disease activity in the ESSDAI domains are summarized in Table 2.

**Table 2:** Numbers and percentages of each active ESSDAI domain at baseline, ever during the follow-up, and at last follow-up visit.

**ESSDAI domains**	**Baseline**	**Cumulative ESSDAI**	**Last follow-up**
	***N* = 508**	***N* = 666**	***N* = 418**
Constitutional, *n (%)*	20 (3.9%)	34 (5.1%)	8 (1.9%)
Lymphadenopathy, *n (%)*	43 (8.5%)	56 (8.4%)	27 (6.4%)
Glandular, *n (%)*	77 (15.1%)	83 (12.5%)	29 (6.9%)
Articular, *n (%)*	130 (25.6%)	156 (23.4%)	82 (19.6%)
Cutaneous, *n (%)*	32 (6.3%)	43 (6.4%)	20 (4.8%)
Pulmonary, *n (%)*	7 (1.4%)	12 (1.8%)	12 (2.9%)
Renal, *n (%)*	3 (0.6%)	4 (0.6%)	6 (1.4%)
Muscular, *n (%)*	0	0	1 (0.2%)
Peripheral nervous system, *n (%)*	8 (1.6%)	25 (3.7%)	23 (5.5%)
Central nervous system, *n (%)*	5 (1%)	5 (0.7%)	5 (1.2%)
Hematological, *n (%)*	58 (11.4%)	99 (14.8%)	77 (18.4%)
Biological, *n (%)*	125 (24.6%)	149 (22.4%)	128 (30.6%)

### Disease Activity at Any Time During the Follow-Up

During the whole follow-up of a mean duration of 9.1 (6.9) years the disease activity fluctuated over time in about one half of the patients. In fact, compared to the baseline ESSDAI score, the onset of new ESSDAI domains and/or worsening of ESSDAI domains already active at baseline was observed in 125/255 (49%) patients, while in 130/255 (51%) patients no change in disease activity was noticed during the follow-up. Disease activity in the ESSDAI domains at any time during the follow-up are summarized in Table 2. The highest disease activity in any time was low in 104/255 (41%) patients, moderate in 85/255 (33%), and high in 66/255 (26%), as shown by the cumulative ESSDAI score. In fact, the median cumulative ESSDAI score was 6 (0–36), i.e., higher than the baseline ESSDAI (median baseline ESSDAI score vs. median cumulative ESSDAI, *p* < 0.0001 by Wilcoxon test). The total number of active cumulative ESSDAI domains during the follow-up was 666. The most frequent active domains were: articular (156/666, 23%), biological (149/666, 22%), hematological (99/666, 15%), and glandular (83/666, 12%).

### Disease Activity at Last Follow-Up

At the last follow-up visit the disease activity was low in 163/255 (64%) patients, moderate in 71/255 (28%), and high in 21/255 (8%). The median ESSDAI score was 3 (range 0–24), significantly lower compared to ESSDAI at diagnosis (*p* < 0.0001) and to the cumulative ESSDAI score in the follow-up (*p* < 0.0001). The total amount of active ESSDAI domains at last follow-up was 418. The most frequent active domains were: biological (128/418, 31%), articular (82/418, 20%), hematological (77/418, 18%), and glandular (29/418, 7%). 41/255 (16%) patients had no systemic involvement (i.e., ESSDAI at last follow-up = 0). Compared to the baseline ESSDAI score, an improvement in ESSDAI disease activity at last follow-up visit was observed in 115/255 (45%), no change occurred in 80/255 (32%), while the onset of new ESSDAI domains and/or worsening of already active ESSDAI domains was shown in 60/255 (23%) patients. Disease activity in the ESSDAI domains at last follow-up are summarized in Table 2.

### Predictive Factors of Long-Term Outcome

Among the baseline ESSDAI domains, both in univariate and multivariate analysis, only the disease activity in articular domain predicted disease activity at the last follow-up visit, being protective in terms of stable or improved disease activity [19/130, 15 vs. 41/125, 33%, *p* = 0.01, by chi square test, OR 2.9 (1.6–5.4)].

During the follow-up, by univariate analysis, the onset and/or worsening of disease activity in the peripheral nervous system (PNS) domain or in the biological domain correlated with a higher level of disease activity at the last follow-up visit [PNS domain: 15/25, 60 vs. 45/230, 20%, *p* < 0.0001 by chi square test, OR 5.9 (2.4–14.5); biological domain: 42/149, 28% vs. 18/106, 17%, *p* = 0.043, by chi square test, OR 1.9 (1.0–3.8)]. By multivariate analysis, only the onset and/or worsening of disease activity in the peripheral nervous system (PNS) domain correlated with a higher level of disease activity at last follow-up visit (*p* < 0.0001, OR 6.1 (2.5–14.9), while the biological domain was no longer selected (*p* = 0.051, OR 1.9 (1.0–3.8).

Finally, the male gender was also associated with a worse outcome at last follow-up by multivariate analysis (*p* = 0.027, OR 3.2 (1.1–9.3).

### Subanalysis of Patients With an Articular Involvement at Baseline

The number of patients showing an active articular ESSDAI domain at diagnosis was 130/255 (50.9%), and the baseline ESSDAI score was significantly higher in patients with articular domain than in those without [5.0, (2–31) vs. 2.0 (0–23), *p* < 0.0001]. A low activity level was registered in 117 patients, a moderate activity level in 12 patients, while a high activity level in 1 patient.

The following variables were significantly associated with the presence of an active ESSDAI articular domain by univariate analysis (Pearson's Chi square test): a lower frequency of anti-SSB antibody (53/130, 40.8 vs. 73/125, 58.4%, *p* = 0.005), a higher frequency of serum cryoglobulins (21/130, 16.2 vs. 3/125, 2.4%, *p* = 0.0002) as well as an overt cryoglobulinemic vasculitis (11/130, 8.5 vs. 2/125, 1.6%, *p* = 0.013), a higher frequency of constitutional symptoms (17/130, 13.1 vs. 3/125, 2.4%, *p* = 0.002), and a higher frequency of cutaneous involvement (22/130, 16.9 vs. 10/125, 8.0%, *p* = 0.032).

In the 130 patients with articular involvement at baseline, also other ESSDAI domains significantly lowered from baseline to the last follow-up (median, range): constitutional (0, 0–6 vs. 0, 0–3, *p* = 0.004, by Wilcoxon log rang test), lymphadenopathy (0, 0–12 vs. 0, 0–12, *p* = 0.03, by Wilcoxon log rang test), glandular (0, 0–4 vs. 0, 0–4, *p* < 0.0001, by Wilcoxon log rang test), articular (2, 2–6 vs. 0, 0–4, *p* < 0.0001, by Wilcoxon log rang test), cutaneous (0, 0–9 vs. 0, 0–6, *p* = 0.004, by Wilcoxon log rang test) and PNS (0, 0–10 vs. 0, 0–10, *p* = 0.004, by Wilcoxon log rang test). More patients with articular involvement were taking hydroxichloroquine (HCQ) at the last follow-up, if compared to pSS patients without articular involvement (41/130, 31.5 vs. 13/125, 10.4%, *p* < 0.0001). The characteristics of the patients with positive articular ESSDAI domain at baseline are reported in Table 3. No difference in all the reported variables was observed (data not shown). The number of patients showing an impairment of the disease activity at the last follow-up was not different between those patients who was taking hydroxychloroquine and those who was not (44/54, 81.5 vs. 151/201, 75.1%, *p* = 0.33, Pearson's chi square test).

**Table 3:** Characteristics of the patients taking hydroxichloroquine at the last follow-up visit.

**Features**	***N* = 54**
Age at diagnosis, years, *mean ± SD*	49 ± 13
Gender, female, *n (%)*	52 (96.3%)
Ethnicity, Caucasian, *n (%)*	100 (100%)
Ocular symptoms, *n (%)*	53 (98.1%)
Oral symptoms, *n (%)*	53 (98.1%)
Positive salivary gland biopsy, *n (%)*	19/24 (79.2%)
Focus score, *mean ± SD*	1.4 ± 0.9
Anti-Ro/SSA positive, *n (%)*	46 (85.2%)
Anti-La/SSB positive, *n (%)*	26 (48.1%)
ANA positive >1:160, *n (%)*	48 (88.9%)
RF positive, *n (%)*	23 (42.6%)
Cryoglobulin positive, *n (%)*	3 (5.6%)
Low C3 levels (<90 mg/dl), *n (%)*	11 (20.4%)
Low C4 levels (<10 mg/dl), *n (%)*	6 (11.1%)
B-cell lymphoma at baseline, *n (%)*	6 (11.1%)
B-cell lymphoma during follow-up, *n (%)*	2 (3.7%)
Baseline introduction of HCQ	35 (64.8%)
Baseline ESSDAI, *median (range)*	5 (0–25)

*RF, rheumatoid factor; ANA, antinuclear antibodies; HCQ, hydroxychloroquine*.

## Discussion

Primary Sjögren's syndrome is a complex disease, still deprived of treatment strategies that may change its natural evolution (18). For this reason, such a natural history of the disease can be still evaluated. To this end, the availability of large cohorts of patients with a long follow-up provides the opportunity to investigate the role of clinical and laboratory features, present either at baseline, or during the disease course, as predictors of the long-term outcome in pSS (19). At present, the ESSDAI composite score represents the main available instrument, validated, and of widespread use, for assessing the systemic activity of the disease (15, 20). The study of large monocentric cohorts with long-term follow-up is also useful to this end. Of note, the pattern of organ involvement in pSS, if compared to ESSDAI, proved to be more relevant to predict one pSS outcome, i.e., lymphoma development, in one recent study (10). Also, novel treatment approaches currently available might be better applied when considering the pattern of organ involvement in pSS, rather than a whole score of disease activity, which may also limit patient recruitment in clinical trials (11).

In the present work, 255 pSS patients were followed in a single Center for over 9 years. Patients were usually evaluated every 6 or 12 months based on the clinical judgement. Overall, systemic disease activity significantly decreased over time in the long term, with around three quarters of patients remaining stable or improving at the last follow-up. In any case, a better disease stratification is needed: patients with a higher risk of a worse outcome must be identified, and an improvement, rather than a disease stabilization, is probably achievable with new treatment options in the future.

We herein highlight the possible disease manifestations which might predict a better or a worse outcome, in terms of a higher disease activity after long-term follow-up if compared to the evaluation at diagnosis: the articular involvement at diagnosis (being protective) and PNS or biologic involvement appeared *ex-novo* or worsening (increasing the risk of deterioration). Importantly, these results are much more robust for the articular and the biologic domains, which were well-represented in our cohort (respectively, in 130 and 125 patients at diagnosis, 156 and 149 patients during the whole follow-up), rather than for PNS involvement, which was observed in only 8 patients at diagnosis, and in a total of 25 patients during the whole observation period.

The sole predictor at diagnosis proved to be the articular domain of the ESSDAI, being protective in terms of stable or improved disease activity level at the last follow-up. Interestingly, the articular manifestations are an indication for the administration of HCQ in our Center. Since not only the articular domain, but also other ESSDAI domains improved in patients suffering from articular manifestations at baseline in this study, it can be hypothesized that HCQ exerts a broader spectrum of beneficial effects in pSS. However, larger studies are needed to this end. In SLE, HCQ is thought to protect against accelerated atherosclerosis targeting toll-like receptor signaling, cytokine production, T-cell and monocyte activation, oxidative stress, and endothelial dysfunction (21). HCQ was also described to have beneficial effects on traditional CV risk factors, such as dyslipidaemia and diabetes (22). Although HCQ, compared with placebo, did not show improvement in symptoms of dryness, pain, and fatigue during 24 weeks of treatment of pSS, evaluation of the long-term outcome under continuous HCQ exposure deserves further controlled studies (23). Secondly, the articular domain was associated with constitutional symptoms, with cryoglobulinemia or with cryoglobulinemic vasculitis, in our cohort. Cryoglobulinemia is a well-established prognostic factor for a worse outcome in pSS (24). Since B-cell depleting therapy was used in some of our pSS patients with articular involvement and cryoglobulinemia, a role of B-cell depleting therapy should be also considered for long-term effects in pSS (25).

When considering the pSS manifestations appearing or worsening during the follow-up, PNS involvement appeared to be linked to an increased level of disease activity at the last follow-up, by multivariate analysis. The low number of patients carrying PNS involvement is a strong limitation for this result, which remains to be definitely confirmed in larger cohorts.

Indeed, PNS is a difficult to treat manifestation in the course of pSS, often leading to a chronic irreversible damage that is usually hardly to distinguish from an active disease (26). Yet, PNS is a common manifestation of cryoglobulinemic vasculitis in pSS, which has been already demonstrated to express a high level of systemic disease activity in pSS, together with a high level of peripheral and local B-cell activation (27). In support of this concept, also the biologic domain of the ESSDAI, when developing or worsening during the follow-up, was significantly associated to a higher level of disease activity at the last follow-up, by univariate analysis. In fact, the biologic domain of the ESSDAI is characterized by some biological markers of B-cell activation, such as the presence of a monoclonal component, hypergammaglobulinemia, cryoglobulinemia, or low complement levels (7).

Again, clinical trials of new treatments for pSS should be focused also on markers of B-cell activation as surrogates of disease subsets at higher risk to develop systemic complications (28).

Taken together, these results point out the importance to consider single clinical manifestations rather than a composite score when the objective is to identify predictive factors of outcome in real-life cohort of pSS, in line with a recently work by our Group putting as an outcome the development of lymphoma (11). Secondly, in real-life cohorts, the ESSDAI score does not follow a normal distribution, the large majority of pSS patients showing a score <5. Fewer patients can be recruited in clinical trials, which usually investigate pSS with ESSDAI >5, and larger populations are necessary to have the adequate power to detect small differences in low-activity disease states.

Finally, a worse outcome was associated to the male gender in the present study. This observation is consistent with recent published work on sex difference in two independent cohorts of incident primary SS, which demonstrated that the presence and number of extraglandular manifestations are significantly more frequent among men than in women with pSS, (29). This study then confirms that pSS males may suffer from a more severe form of disease.

To conclude, articular involvement at diagnosis and the development or worsening of PNS during the follow-up are important clinical features for predicting the outcome in pSS. PNS involvement may represent a clinical manifestation to be followed more strictly and, probably, to require an appropriate treatment. In addition, the biologic involvement and the evaluation of biologic disease activity deserves additional study in pSS, in our opinion. The use of HCQ as backbone treatment in the long term still needs a further evaluation. Overall, a clinical approach mainly considering the single organ manifestations in pSS, rather than a composite score of disease activity alone, appears worthwhile (10).

## Ethics Statement

This study was carried out in accordance with the recommendations of local ethic committee, named Comitato Etico Unico Regionale (C.E.U.R.) with written informed consent from all subjects, as required for the collection of clinical data in the HARMONICSS project. All subjects gave written informed consent in accordance with the Declaration of Helsinki. The protocol was approved by the Comitato Etico Unico Regionale (C.E.U.R.).

## Author Contributions

LQ and SD conceived of the presented idea. LQ developed the hypothesis and performed the analyses. SD verified the analytical methods. SD encouraged LQ to investigate specific aspects of the research and supervised the findings of this work. SG, AZ, SZ, and CF collected the data and provided support for the development of the hypothesis. All authors discussed the results and contributed to the final manuscript.

### Conflict of Interest Statement

The authors declare that the research was conducted in the absence of any commercial or financial relationships that could be construed as a potential conflict of interest.
